# Hematopoietic Cell Transplantation for Sickle Cell Disease

**DOI:** 10.3390/children13060741

**Published:** 2026-05-26

**Authors:** Gregory Michael Taylor Guilcher

**Affiliations:** Departments of Oncology and Pediatrics, Cumming School of Medicine, University of Calgary, Calgary, AB T2N 4N1, Canada; greg.guilcher@ahs.ca

**Keywords:** sickle cell disease, hematopoietic cell transplantation, transplant, outcomes, late effects

## Abstract

**Highlights:**

**What are the main findings?**

- Sickle cell disease affects millions of people globally, and increasing numbers of patients are seeking curative therapies.

**What are the implications of the main findings?**

- Allogeneic hematopoietic cell transplantation is a well-established curative therapy for the minority of patients with a matched sibling donor;
- Alternative donor transplantation is becoming safer; however, graft-rejection and graft-versus-host disease limit its use with caution to those with severe phenotypes.

**Abstract:**

Sickle cell disease is the most common monogenetic disease worldwide and affects millions of children and adults. While supportive care practices have improved life expectancy, particularly in high income countries, sickle cell disease typically has adverse impact on quality of life and reduced life expectancy. As a result, patients and providers are increasingly seeking information regarding curative and transformative therapies and advocating for improved global access. This review will describe allogeneic hematopoietic cell transplantation eligibility, approaches to transplant, short and long-term outcomes and key supportive care considerations for providers who care for patients with sickle cell disease.

## 1. Introduction

Hematopoietic cell transplantation (HCT) has been an established cure for sickle cell disease for over 40 years (SCD) [1,2]. While there have been advances in supportive care practices and disease-modifying agents, even in high income countries, life expectancy remains shorter than those without SCD, and this chronic vasculopathy can have significant impact on quality of life [3,4,5]. Many patients and families want a safe and effective curative option as a result, but given the potential risks of HCT shared decision-making can be complex [5,6,7,8]. Cost and donor-availability are some of the important reasons why access to HCT has been limited in both high income and resource limited settings. For patients for whom HCT has been offered, risks of treatment-related mortality, graft-versus-host disease, infertility and other long-term effects of HCT remain important factors in decision-making.

Fortunately, there have been many advances in curative and transformative therapies over these decades and notably over the last ten years [8,9]. The number of transplants has increased steadily and transplant programs have been established in low and middle-income countries [2,10,11,12,13,14]. This review will summarize the key literature describing outcomes of HCT for SCD with a focus on the role of the type of donor and stem cell source in high income settings. Established eligibility criteria will be presented with a discussion of who should be considered for HCT and the optimal timing based on recipient disease severity and age. Supportive care considerations, second malignant neoplasms and late effects will be reviewed. Finally, future directions to improve safe access to HCT globally and the relationship with other curative and transformative options such as gene therapy options will be considered.

## 2. Indications for Allogeneic Hematopoietic Cell Transplantation

There is no single validated disease severity classification for SCD, although several practical scores and eligibility criteria have been developed, some specifically developed to guide HCT clinical trial inclusion criteria [15,16,17,18]. To summarize, common eligibility criteria include recurrent vaso-occlusive events (VOEs) over several years prior to HCT despite optimal medical management (e.g., hydroxyurea), recurrent acute chest crises, neurovascular disease or alloimmunization, as examples. Hemoglobinopathy physicians and transplant physicians are more likely to recommend HCT than those who are less familiar with the notable lifetime risks of SCD (especially into adulthood) and the evolving improvements in HCT practices for this disease [19]. A history of stroke or moyamoya is generally agreed upon as a strong indication for HCT. With excellent outcomes for matched sibling donor HCT, some programs will offer HCT for less severe phenotypes. Younger patients have the fewest HCT complications but may not have had as many SCD complications as a result of their young age [20]. Alternative donor HCT is almost always reserved for children and adolescents with more severe disease courses, often neurovascular disease, recurrent life-threatening acute chest crises or alloimmunization, as examples.

Parents and providers want reassurance that the risks of HCT are justified but also want to prevent severe or life-threatening complications due to SCD which may be irreversible (e.g., stroke) [21,22]. Evolving data on the potential neuroprotective effect of HCT may inform this shared decision making as data emerge [23]. Other organ function may also be preserved, which is important to consider in this discussion [24,25,26,27]. The section Long-Term Follow-Up and Outcomes will discuss this evolving field further.

## 3. Matched Sibling Donor Allogeneic Hematopoietic Cell Transplantation

HCT with a matched sibling donor (MSD) is the most established curative therapy for SCD [2,14]. Rates of disease-free survival exceed 90% in many landmark studies with lower risks of GVHD compared to alternative donor HCT, as summarized in Table 1 and Table 2 [28,29,30,31,32,33,34,35,36,37,38,39,40,41,42,43,44]. Rates of acute GVHD and chronic GVHD vary, but typically range from 15–20% and 10–15%, respectively. Unfortunately, only 15–20% of patients will have an MSD who is also unaffected by SCD. For those who do have an available MSD, most practitioners require some disease complications before offering HCT, with criteria for eligibility varying by country and institution [14,15,19,20]. With clearly better outcomes in younger patients (best outcomes under 5 years of age, clearly worse outcomes for those older than 13 years), the balance between requiring an established history of sickling complications for eligibility and proceeding to HCT at an age when HCT outcomes are maximized is not always clear [19,20,21]. Some HCT programs will offer allogeneic transplant to any patient with an MSD, even if minimally symptomatic.

## 4. Unrelated Donor Allogeneic Hematopoietic Cell Transplantation

Outcomes with unrelated donor HCT have historically been suboptimal, with higher rates of GVHD and resultant mortality [21,22]. An international study of reduced-intensity conditioning and unrelated donor HCT had unacceptably high rates of GVHD [34]. However, as GVHD prevention strategies have advanced, more unrelated donor transplants are being performed for patients with severe phenotypes [11]. The use of abatacept and reduced intensity conditioning has reduced rates of acute and chronic GVHD in two small pilot studies [35,36]. The use of post-transplant cyclophosphamide for in vivo T-cell depletion has also been studied in a small number of patients to mitigate GVHD risk [45,46]. Based on existing data, most experts will limit the use of unrelated donor HCT to those with severe phenotypes, notably neurovascular disease.

While improving outcomes of unrelated donor HCT will benefit some patients with SCD, it must be emphasized that most patients with SCD will not find a fully HLA-matched donor on existing unrelated donor registries, although mismatched donors are available at higher frequencies [47,48]. Expanding access to safe unrelated donor HCT will require strategies which can mitigate risks of GVHD in mismatched unrelated donors.

## 5. Haploidentical Allogeneic Hematopoietic Cell Transplantation

The use of haploidentical HCT has greatly expanded access to HCT for many patients with diverse indications for transplant around the world [49]. A significant advantage of haploidentical HCT is that almost every person will have a donor. While initial studies of haploidentical HCT have had unacceptably high rates of graft failure, results of haploidentical HCT in adults using post-transplant cyclophosphamide are very encouraging, with high rates of engraftment and rates of GVHD that are comparable to those with an MSD [38,44]. Strategies to mitigate risks of graft rejection have included increasing the dose of total-body irradiation or the addition of thiotepa to reduced-intensity conditioning regimens [40,50]. Unfortunately these strategies have yet to achieve the same results in patients under 18 years of age that have been published in adults [38,39]. More intensive strategies using myeloablation have yielding encouraging results in children and adolescents in pilot studies, but this approach has not been widely adopted [37]. International efforts led by European colleagues to mitigate risks of graft rejection and GVHD using peripheral blood stem cell (PBSC) allografts and various techniques for ex vivo T-cell depletion are underway (NCT04201210) [51].

## 6. Conditioning Regimens

The first transplants for SCD were performed with busulfan-based myeloablative conditioning [1,28,29,52,53]. Long-term follow up data describing outcomes of myeloablative conditioning with busulfan and the inclusion of anti-thymocyte globulin (ATG) have established this conditioning approach as the gold-standard according to the American Society of Hematology (ASH) for children with SCD [15,30]. The French experience is impressive with a five year event-free survival (EFS) of 98% and a treatment-related mortality risk under 2% [30,53].

While this approach remains the international standard for children, challenges remain and require ongoing efforts to reduce acute and late toxicities for people with SCD of all ages [14,15,54]. In particular, outcomes with myeloabative conditioning in older adolescents and adults remain poor, and ASH recommends nonmyeloablative approaches to reduce the risks of TRM and GVHD in this population at higher risk for these complications [14,21,22]. A nonmyeloablative regimen developed at the National Institutes of Health (NIH) using alemtuzumab and low dose total body irradiation (TBI) with rates of DFS over 80% and negligible risks of GVHD have resulted in its adoption in many countries for adults, although many adult HCT programs still do not offer HCT for SCD [42,43,55]. Recent concerns regarding clonal hematopoiesis for adult patients with graft rejection and mixed donor myeloid chimerism have resulted in caution with this approach, and efforts are ongoing to refine this regimen to increase donor myeloid chimerism [56,57]. Second malignant neoplasms will be discussed later in this review.

Despite outstanding results with myeloablative busulfan in children with SCD and an MSD, practitioners are still exploring regimens which may reduce the undesired short and long-term effects of intensive chemotherapy [8,58]. One of the most established reduced intensity conditioning regimens involves the use of alemtuzumab, melphalan and fludarabine [32,58,59]. Rates of GVHD are comparable to myeloablative busulfan, and overall and disease-free survival are over 90% in clinical trial and real-world registry reports [59]. Reduced intensity and nonmyeloablative regimens may have less impact on health-related quality of life in the short-term, require shorter hospitalizations and might be less costly [8]. Another important goal is to reduce gonadotoxicity and rates of infertility, which are high with myeloablative busulfan and a complication that is highly undesirable [60,61]. However, despite the aim of reducing gonadotoxicity, the use of reduced intensity conditioning regimens have yet to achieve this goal [61]. The important issue of infertility is reviewed later in this manuscript.

Finally, several nonmyeloablative conditioning regimens have also been studied in pediatric SCD, including the NIH regimen which was developed for adults. However, in an international multi-center study of this regimen and Center for International Blood and Marrow Transplant Research real-world data rates of graft failure between 10 and 20% preclude its adoption for routine use in children despite the near absence of GVHD [33,59]. The “Minimizing Toxicity in HLA-Identical Sibling Donor Transplantation for Children with Sickle Cell Disease” or “SUN” study is ongoing, with amendments to reduce the risk of graft rejection [33]. Table 1 and Table 2 summarize landmark publications in HCT for SCD with various conditioning approaches by donor type for pediatric and adult recipients, respectively.

## 7. Stem Cell Source

The vast majority of transplants for SCD have been with an MSD bone marrow allograft [2,14]. Rates of GVHD with haploidentical bone marrow allografts using post-transplant cyclophosphamide have been comparable to rates of GVHD with an MSD and bone marrow, although in patients under 18 years of age risks of graft rejection remain a barrier and deaths due to GVHD still occur [38,44]. The risks of GVHD and mortality have been documented to be higher with peripheral blood stem cells (PBSCs) and lower with umbilical cord blood (UCB), although the risks of graft failure are higher with the latter [2,62,63,64,65,66]. Despite these challenges, the ASH recommendations *suggest* the use of MSD UCB provided the cell dose is adequate [14]. UCB can be combined with marrow from an MSD if the cell dose of the cryopreserved UCB product is insufficient. Myeloablation should be used with UCB as a stem cell source.

With respect to PBSCs, it should be noted that while the risks of GVHD are well established to be higher with PBSCs, when in vivo or ex vivo T cell depletion is used, this risk can be mitigated [33,67,68]. With the use of PBSCs and the NIH nonmyeloablative regimen, the risk of acute and chronic GVHD is almost negligible [33,42,43,67]. An international study of PBSCs with ex vivo T cell receptor (TCR) αβ/CD19 depletion is underway (NCT04201210) [51].

## 8. Graft-Versus-Host Disease and Its Prevention

GVHD remains one of the most important causes of morbidity and mortality in HCT for SCD [22]. In addition to established risk factors such as donor (other than an MSD), degree of HLA matching and stem cell source, there are in vivo and ex vivo measures that can be taken to reduce this risk. Examples of in vivo strategies include optimizing ATG dosing, the use of alemtuzumab as serotherapy, post-transplant cyclophosphamide and the use of newer medications such as abatacept [35,36,45,46,69,70]. A Sickle Cell Transplant Advocacy and Research Alliance (STAR) study in development will adapt an approach combining abatacept and post-transplant cyclophosphamide used in adults to spare patients calcineurin inhibitors and the associated risks (e.g., nephropathy and posterior reversible encephalopathy syndrome, or PRES), some of which are greater in patients with SCD [70]. Studies employing ex vivo graft manipulation with either CD3 or TCRab/CD19 depletion are underway in Europe and show promise to reduce rates of severe GVHD and safely expand both matched and mismatched allogeneic HCT [51]. Other investigators have controlled CD3 graft content by enriching the allograft for CD34+ cells with prescribed mononuclear cell addback [37]. Strategies to prevent GVHD will improve access to HCT for more patients with SCD, and are critical to increase the safety of alternative donor transplantation.

## 9. Mixed Donor Chimerism

Several studies have shown that the sickle phenotype can be reversed with less than full donor chimerism, even as little as 20–25% donor [71,72]. As a result, less intensive conditioning regimens have been evaluated to minimize acute and late toxicities, and increase the number of patients eligible for HCT. However, mixed donor myeloid chimerism can predict lower chimerism over time, and with emerging concerns over clonal hematopoiesis and treatment related malignancies many experts advocate for regimens which maximize the likelihood of full donor myeloid chimerism [56,57,73]. See the later section Second Malignant Neoplasms and Clonal Hematopoiesis for further discussion of this very important issue.

It should be noted that even with an MSD and myeloablative conditioning, mixed donor chimerism is common [30]. Donor T-cell chimerism appears less relevant to long-term graft outcome [73,74].

Historically whole blood chimerism analysis was the most common method of evaluating donor engraftment and it is important to highlight the assays used when reviewing the existing literature [72]. Lineage-specific evaluation of myeloid chimerism has since become best practice [73,75,76]. Strategies to better evaluate erythroid engraftment specifically have yet to become standard of care [77]. Hemolysis can be seen when donor chimerism is less than 50%, typically closer to 25% donor myeloid chimerism [30,73].

A common clinical dilemma encountered in the setting of dropping or low mixed donor chimerism is if, when and how to intervene. Options include increasing immune suppression (with risk of toxicity), reducing or withdrawing immune suppression (with risk of GVHD) or additional cell infusions/second transplants. Further complicating this decision-making process is the possible risk of treatment-related malignancy with ongoing stress hematopoiesis and the donor availability for additional donations, particularly if the donor is a minor [78,79]. Case series describe improvement in donor myeloid chimerism when lymphodepleting chemotherapy and serotherapy is given, with some experts advocating for donor chimerisms approaching 20% before intervention [80,81,82,83]. Donor lymphocyte infusions are generally discouraged due to the risk of GVHD in a non-malignant condition.

## 10. Supportive Care

There are unique supportive care measures for patients with SCD who undergo HCT, many originating from the landmark clinical trial 30 years ago [84]. These include specific transfusion medicine thresholds such as achieving a hemoglobin S level under 30–40% prior to conditioning (via simple or exchange transfusion), maintaining post-HCT hemoglobin levels of at least 80–90 g/L using simple transfusions and platelets over 50 × 10^9^/L to prevent sickling crises and intracranial hemorrhages. Patients with SCD are at higher risk of PRES, so seizure prophylaxis is required with calcineurin inhibition (cyclosporine and tacrolimus) and attention to avoiding hypertension and maintaining normal magnesium levels [85]. Anti-epileptic drugs are not required with mTOR inhibitors such as sirolimus. Monitoring for transplant-associated thrombotic microangiopathy (TA-TMA) is also very important. Pencillin prophylaxis continues (or is reintroduced) until re-vaccination against pneumococcus has been completed post-HCT, and some centers perform studies of splenic function before discontinuation as recovery of splenic function is variable and less likely in older patients (Table 3) [8,23,24,25,26,27,52,86,87,88,89,90,91,92,93,94,95,96,97,98,99,100]. Granulocyte colony-stimulating factor is used in some protocols to hasten neutrophil recovery and has been established to be safe post-HCT. While some of these practices may be questioned based on the level of evidence supporting their necessity, they remain best practice based on expert opinion and the low risk tolerance for major toxicities in this patient population.

## 11. Psychosocial Supportive Care

HCT is an intensive medical and psychological experience with notable risks, as described above. It is essential to provide psychological support for any person undergoing HCT, but there are additional considerations for those with SCD [75,85,101]. It is worth highlighting that some survivors post-HCT experience identity conflict as they transition from being a “Sickle Cell Warrior” to a person who is “cured,” sometime leaving behind not only an identity but a sickle cell community of support. The need for adequate resources and trained professionals to provide pro-active support patients and caregivers should not be underestimated.

## 12. Fertility Preservation

Sickle cell disease and disease modifying agents can impact gonadal function [102,103]. Impaired blood flow to the gonads, priapism in males, iron overload, impaired physical and social functioning and hydroxyurea can all impact fertility outcomes even without HCT conditioning. HCT is well known to affect fertility; fertility preservation is highly important to any HCT recipient, and also of high importance to patients with SCD and their families [60,104]. Reduced reproductive potential and especially missed opportunities for fertility preservation have major implications for long-term quality of life. While barriers exist to access standard of care interventions such as sperm banking, oocyte and ovarian tissue cryopreservation, such barriers should not preclude referral to an expert in this field for consultation [105,106]. Even when such discussions take place, not all survivors recall the content of the discussion, highlighting opportunities for improvement in patient education and emphasizing the longitudinal approach required [60]. Patients and families may still choose to undergo HCT with risks of reduced fertility, but many experts and advocates believe that accessing the most current best practices and reducing barriers are ethical imperatives [105,106]. While nonmyeloablative conditioning regimens may improve fertility outcomes, myeloablative and reduced-intensity conditioning are associated with high rates of gonadal dysfunction [30,33,60,61,106,107,108]. Specialist referral post-HCT is also important as some patients may benefit from assisted reproductive care or may have a reproductive window that may be shorter compared to those who have not undergone HCT [109].

## 13. Second Malignant Neoplasms and Clonal Hematopoiesis

The first transplant for SCD was performed primarily for acute myelogenous leukemia [1]. While some studies suggest that people with SCD are at increased risk of clonal hematopoiesis, the absolute risk remains low in the absence of genotoxic exposures (e.g., high dose chemotherapy or radiation) [110]. A larger international registry-based study demonstrated a 10-year incidence of any malignancy of 2.4% and of leukemia/MDS of 1.7% [111]. Solid tumors and myelodysplastic syndrome/acute myelogenous leukemia were noted in 5 patients ≤ 18 years of age at transplant, respectively. Of those with myeloid malignancy, 2/5 had TBI and the remaining patients had only chemotherapy exposure. All the adult recipients who developed myeloid malignancies had TBI, reflective of the most common conditioning approaches in older patients with SCD. Most (but not all) patients who develop myeloid malignancies have graft failure, but there are reported cases in patients with mixed donor chimerism. A description of two nonmyeloablative regimens at the National Institutes of Health documented 8 patients with hematologic malignancies, 6 of whom had myeloid malignancies [112]. Of these 8 patients, 5 had graft failure.Clonal hematopoiesis develops increasingly with age, and years of stress hematopoiesis or regenerative hematopoiesis and inflammation in the setting of graft rejection or poor engraftment may increase this risk further [56,57]. While patients who develop myeloid malignancy after allogeneic HCT typically have TP53 mutations, the two cases after busulfan exposure and autologous gene therapy had *RUNX1* and *PTPN11* mutations with monosomy 7 [57]. Baseline mutations have been documented in several patients, albeit at much lower levels than after HCT. While much remains to be learned about the risk in children compared to adults, the role of mixed donor chimerism and rates of myeloid malignancy in patients who undergo busulfan-based autologous gene therapy in greater numbers, most experts agree that achieving high donor myeloid chimerism and avoiding graft failure are priorities [57,112].

## 14. Long-Term Follow-Up- Beyond Survival and GVHD

Studies of HCT outcomes across the many indications for transplant often focus on overall survival, graft-rejection and rates of GVHD [113]. However, more detailed and nuanced measures of success are needed in SCD. Many experts counsel patients that HCT might stop acute sickling crises and prevent ongoing end organ damage, but that existing damage is not expected to reverse. Chronic pain and avascular necrosis, as examples, often persist post-HCT [113,114].

However, data is emerging to address this knowledge gap. The largest registry of sickle cell specific data from the STAR consortium is actively describing the functional outcomes of several organ systems post-HCT [24]. The European Society for Blood and Marrow Transplantation Haemoglobinopathy Working Party has a similar registry [115]. A summary of some of the key studies of late effects are summarized in Table 3. Existing data suggest that neurovascular, pulmonary, cardiovascular and renal disease largely stabilize, with some studies demonstrating improvement in some parameters for each organ system.

Critical information for patients, families and providers is the health-related quality of life (HRQOL) and neurocognitive outcomes post-HCT. There are some data describing such outcomes, but overall more high quality studies are needed [23,116,117,118]. Data suggesting that the anticipated decline in neurocognitive function with SCD may be stabilized is highly relevant to decision-making. To address these important knowledge gaps, prospective studies such as Project Sickle Cure (STAR) and WeDecide (a STAR collaboration with the Globin Regional Data and Discovery registry) are underway [7,118]. Early data from the Project Sickle Cure study demonstrate remarkable improvement in HRQoL in multiple domains, and low rates of decisional regret.

## 15. What About Second HCT?

There are limited data on the outcomes of second HCT for SCD. However, data from North America and Europe have emerged very recently [115,119]. The rates of overall and disease-free survival are lower than those published for first HCT, prompting caution when counselling patients and families. The North American data showed no effect of conditioning intensity, although a short interval between the two transplants showed a markedly reduced event-free survival, likely reflective of marrow aplasia after graft rejection and the increased toxicity with a short period between conditioning regimens. More data is needed to inform standardized recommendations.

## 16. How Does Gene Therapy Change the Field of Allogeneic HCT?

Autologous gene therapy is commercially available in several countries around the world, using lentiviral and CRISPR-CAS9 technology [120,121,122]. Base-editing strategies are under study, and in vivo treatment holds great hope to reduce toxicity and improve access to cure [123,124,125]. Advantages of gene therapy include the absence of GVHD and more donor-availability, although not every patient is eligible for this treatment nor can all achieve successful mobilization or infusion. The current high cost of this treatment is a key consideration in all parts of the world and makes this therapy prohibitive in regions where the disease is most prevalent. While a review of gene therapy approaches is beyond the scope of this review, the roles of disease-modifying agents, allogeneic HCT and gene therapy are in constant evolution and must all be positioned against each other in providing the best treatment information to patients and families. The underlying phenotype might inform this decision making- for example, outcomes of gene therapy for patients with neurovascular disease are less well established. Alternatively, haploidentical HCT may not be appropriate in a patient with significant donor specific antibodies. In an effort to provide consistent information and recommendations nationally in Canada, Cell Therapy Transplant Canada and the Canadian Haemoglobinopathy Association (CanHaem) have partnered and published a co-created practice guideline [126]. These recommendations largely align with those of the EBMT. As new data become available, these guidelines will require updating to ensure that providers can provide the most current information and recommendations to their patients. In high income countries, gene therapy is a major addition to curative therapeutic options [125,127].

## 17. Expanding Global Access to Regions in Greatest Need

Allogeneic HCT is a costly treatment and requires notable infrastructure [13,125]. While HCT may be cost saving compared to a lifetime of disease-modifying therapy or gene therapy, barriers to access exist within high income countries (such as provider biases, information provided to patients and insurance, as examples) [19,85,127]. Cost barriers and lack of infrastructure make HCT even more inaccessible in the countries with the greatest burden of disease [125]. However, there are established HCT programs for SCD in Sub Saharan Africa which are active with high rates of success, some comparable to those of high income countries [10,13]. The EBMT has established a subgroup within its Haemoglobinopathy Working Party, and meaningful partnerships are being created between programs in high and low-middle income countries. Such partnerships are essential to truly build capacity and access to cure to the regions in most need, and it is paramount that self-sustainable expertise and infrastructure be established, led by the providers practicing in those regions.

## 18. Future Directions: Challenges and Opportunities

A marked increase in the number of allogeneic HCTs performed globally over the last decade attests to the improvement in safety and outcomes over the last 40 years. As the risks of mortality and GVHD are reduced, the more acceptable this treatment will be to patients and providers. In addition, more patients and families are requesting discussions around curative and transformative therapies as access to information and patient/provider education efforts improve.

Access to allogeneic HCT is largely currently limited to those with an MSD. Expanding donor access is critical, largely through improving outcomes with haploidentical HCT. Outcomes with haploidentical transplantation are improving notably in adults with SCD, but graft rejection remains an issue for children. All allogeneic HCT approaches will benefit from reduction or elimination of GVHD. Conditioning regimens which are minimally gonadotoxic are highly important to patients; with myeloablative conditioning the current standard for children as per the American Society of Hematology and the EBMT, the risks of infertility remain an important issue. Even with NMA, adult patients can have notable fertility challenges based on a small body of literature [109]. While fertility preservation strategies exist, they are not available as standard of care to all patients (e.g., prepubertal males) and come with some medical and financial toxicities. A better understanding of the long-term outcomes of HCT is still needed- does HCT improve life expectancy? What are the long-term impacts on quality of life and organ function after decades?

Lastly, access must be considered through a global lens, whereby cure can be offered to all patients with SCD regardless of the country or region in which they live.

## Figures and Tables

**Table 1:** Outcomes of allogeneic hematopoietic cell transplantation for children and adolescents with sickle cell disease by donor type and conditioning intensity.

	Authors	N	Median Age (Years)	Conditioning	Overall Survival	Graft Rejection	Acute GVHD	Chronic GVHD	TRM
**Matched Sibling Donor**									
*Myeloablative*									
	Walters et al. [28]	50	9.4	Bu/Cy/ATG	94%	10%	15%	12%	6%
	Dedeken et al. [29]	50	8.3	Bu/Cy ± ATG	94%	8%	22%	20%	<5%
	Bernaudin et al. [30]	195		Bu/Cy ± ATG	96%	3%	21% *	11%	<5%
	Bhatia et al. [31]	18	8.9	Bu/Flu/ Alemtuzumab	100%	0%	17% *	11%	0%
*Reduced Intensity/* *Nonmyeloablative*									
	King et al. [32]	43	13	Flu/Mel/ Alemtuzumab	93%	0% **	31%	13% *	7%
	Nickel et al. [33]	38	13.5	300 cGy TBI/Alemtuzumab	100%	18%	0%	0%	0%
**Unrelated Donor**									
*Reduced Intensity*									
	Shenoy et al. [34]	29	14	Flu/Mel/ Alemtuzumab	73%	10%	28% *	62%	27%
	Shah et al. [35] #	16 ***	12	Flu/Mel/ Alemtuzumab ± TT	95%	10%	20%	35%	5%
	Chaudhury et al. [36]	23	11	Flu/Mel/ Alemtuzumab/TT	100%	100%	4% *	22%	0%
**Haploidentical**									
*Myeloablative*									
	Cairo et al. [37]	19	13	Bu/Cy/Flu/ATG/TT/TLI	84%	0%	6% *	7% *	16%
*Reduced Intensity*									
	Kassim et al. [38]	32	_	Cy/Flu/ATG/TT/200 cGy TBI	94%	25%	8% *	19% *	6%
	Walters et al. [39]	41	12.5	Cy/Flu/ATG/TT/ 200 cGy TBI	95%	15%	16% *	30%	5%
	Goldenberg et al. [40]	16	12	Cy/Flu/ATG/ 400 cGy TBI	100%	6%	25%	25%	0%

* Only high grade acute and/or chronic GVHD reported. # Some data combined with recipients with thalassemia. ** Two patients had donor lymphocyte infusions. *** Only abatacept cohort included. GVHD—graft versus host disease; TRM = treatment-related mortality; Bu = busulfan; Cy = cyclophosphamide; ATG = anti-thymocyte globulin; Flu = fludarabine; cGy = centigray; TBI = total body irradiation; TT = thiotepa; TLI = total lymphoid irradiation.

**Table 2:** Outcomes of allogeneic hematopoietic cell transplantation for adults with sickle cell disease by donor type and conditioning intensity.

	Authors	N	Median Age (Years)	Conditioning	Overall Survival	Graft Rejection	Acute GVHD	Chronic GVHD	TRM
**Matched Sibling Donor**									
*Myeloablative*									
	Krishnamurti et al. [41]	17	32 *	Bu/Flu/ATG	94%	6%	18% **	29%	6%
*Nonmyeloablative*									
	Inam et al. [42]	62	28.5	Alemtuzumab/ 300 cGy TBI	93%	15%	0%	0%	7%
			29	Pentostatin/Cy/ Alemtuzumab/ 300 cGy TBI	90%	7%	7%	0%	10%
	Alzahrani et al. [43]	122	29	Alemtuzumab/ 300 cGy TBI	94%	13%	2%	0%	4%
**Haploidentical**									
*Reduced Intensity*									
	Kassim et al. [38]	38	25	Cy/Flu/ATG/TT/ 200 cGy TBI	92%	0%	10%	10%	9%
	Kassim et al. [44]	42	23	Cy/Flu/ATG/TT/ 200 cGy TBI	90%	7%	26% *	22%	10%
	Goldenberg et al. [40]	27	27	Cy/Flu/ATG/ 400 cGy TBI	93%	4%	19%	7%	4%

* Entire cohort of matched related and unrelated donors. ** Only high grade acute and/or chronic GVHD reported. GVHD—graft versus host disease; TRM = treatment-related mortality; Bu = busulfan; Flu = fludaraine; ATG = anti-thymocyte globulin; cGy = centigray; TBI = total body irradiation; Cy = cyclophosphamide; TT = thiotepa.

**Table 3:** Late effects outcomes for allogeneic HCT for sickle cell disease.

Organ/System	Authors	N	Cohort Age at HCT	Median Duration of F/U (Years)	Description of Cohort	Key Findings
*Spleen*						
	Nickel et al. [86]	90	Children and Adolescents	2	Retrospective 92% had MAC	Improved splenic uptake on liver-spleen scans Less improvement in older patients cGVHD associated with absent uptake
	Vermylen et al. [52]	10	Children, adolescents and young adults	13	Retrospective	Tc-99m-RBC and Cr-51 damaged red cells 7 had increase in splenic red cell pool with 2 showing improved phagocytosis of damaged red cells 3 had no improvement (2/3 had cGVHD, 1/3 was 17 years at HCT)
*Pulmonary*						
	Walters et al. [87]	23 (with pre/post HCT testing)	Children and Adolescents	NR	Clinical trial with supplemental F/U	Largely stable findings in restrictive and obstructive disease and DLCO Significant decrease in RV and RV/TLC
	Horan et al. [27]	130	Children and Adolescents	3.5	Retrospective	Stability in multiple intrapatient parameters
	Monagel et al. [26]	12	Children and Adolescents	2.6	Retrospective 100% had NMA	Stability in FVC, FEV1, FEV1/FVC Improvement in FEF25–75
	Friedman et al. [88]	19	Children and adolescents	NR, 11 had 2 years of F/U	Retrospective 100% had MAC	Stability in FVC, FEV1, FEV1/FVC, TLC, DLCO Improvement in specific airway conductance
	Ruhl et al. [89]	97	Adults	NR, 55/97 had 3 years of F/U	Retrospective 100% had NMA	Stability in multiple parameters Improvement in DLCO and 6MWD
*Cardiac*						
	Friedman et al. [88]	17	Children and adolescents	NR, 15/17 had 2 years of F/U	Retrospective 100% had MAC	No pulmonary HTN or LV systolic dysfunction pre or post-HCT
	Sukkar et al. [90]	228	Children and adolescents	3.4	Retrospective 76% had MAC	Modest decrease in EF MV flow, TV flow, TRJV all decreased significantly 93% without cardiac dysfunction
	Limerick et al. [91]	12	Adults	1 year post-HCT outcomes reported	Retrospective 100% had NMA	Improvement in cardiac morphology Significant improvement in mean TRJV with mean normalization
*Renal*						
	Pederson et al. [25]	18	Children and adolescents	0.35	Retrospective 100% had NMA	No AKI Long-term HTN in 6/18 Significant reduction in GFR post-HCT, likely due to improvement in hyperfiltration eGFR > 90/mL/min/1.73 m^2^ in all at last follow-up
	Limerick et al. [92]	95	Adults	3 year post-HCT analysis reported	Retrospective 100% had NMA	AKI often noted early post-HCT Overall stable renal function at 3 years post-HCT Reduction in GFR may represent reduction in hyperfiltration
*Brain*						
	Walters et al. [87]	46	Children and adolescents	Varied by outcome	Clinical trial with supplemental F/U	26/26 patients with stroke and 8/10 with silent cerebral infarction and donor engraftment had improved imaging Overall stable cerebral vasculopathy
	Aumann et al. [93]	14	Adults	1.1	Prospective comparison of patients on HU, post-HCT or Hb altering treatment	Improvement in Hb and cerebral blood flow, increase in venous relaxation rate
	Stenger et al. [24]	159	Children and adolescents	2.1	Retrospective	Normal or unchanged MRI post-HCT in 79% New or progressive infarcts (8.2%) or vasculopathy (7.6%) Other new finding in 10.7%
	Carpenter et al. [94]	44			Retrospective Patients with pre-HCT stroke/vasculopathy	No progression of vasculopathy post-HCT 2 strokes and 2 SCIs seen post-HCT in patients with prior ischemic injury
	Verlhac et al. [95]	28	Children and adolescents	3 years F/U	Prospective randomized trial compared to best supportive care	No ischemic infarcts in either group Significant improvement in stenosis scores HCT group and better prevention of new stenoses
	Vermylen et al. [52]	50	Children, adolescents and young adults	NR	Retrospective	No new strokes, including 4 patients with strokes prior to HCT
	Al-Jefri et al. [96]	25	Children and adolescents	4.3	Retrospective 100% had MAC	Many had pre-HCT stroke or moyamoya 8 required long-term anti-epileptic drugs 11 had clinical improvement in hemiparesis
	Hulbert et al. [97]	10	Children	F/U at 1-2 years post-HCT	Prospective comparative study 100% had RIC	Improved cerebral hemodynamics post-HCT compared to non-SCD controls
	Thurn et al. [98]	13	Children, adolescents and adults	0.3	Retrospective	TAMMV normal or unchanged in 7 with post-HCT measurements
	Fay-McClymont et al. [23]	9	Children and adolescents	1.4	Retrospective 100% had NMA	Mean FSIQ was average pre and post-HCT
	Braniecki et al. [99]	14	Children and adolescents	F/U at 1 and 2 years post-HCT (12/14 had all time points)	Prospective clinical trial 100% had MAC	Stable neuroimaging and neurocognitive (DIVERGT) testing post HCT
	Carlson et al. [100]	22	Adolescents and adults	F/U at 1 year	Prospective 100% NMA	Stable neurocognitive function Possible improvement in processing speed

F/U = follow-up; MAC = myeloablative conditioning; cGVHD = chronic GVHD; NMA = nonmyeloablative conditioning; FVC = forced vital capacity; FEV1 = forced expiratory volume in 1 s; FEF25–75 = forced expiratory flow from 25% to 75% of the vital capacity; DLCO = diffusing capacity of the lung for carbon monoxide; 6MWD = Six-minute-walk distance; TLC = total lung capacity; HTN = hypertension; LV = left ventricle; HCT = hematopoietic cell transplantation; EF = ejection fraction; MV = mitral valve; TV = tricuspid valve; TRJV = tricuspid regurgitant jet velocity; RIC = reduced intensity conditioning; AKI = acute kidney injury; GFR = glomerular filtration rate; eGFR = estimated glomerular filtration rate; HU = hydroxyurea; MRI = magnetic resonance imaging; SCI = silent cerebral infarction; Hb = hemoglobin; RIC = reduced intensity conditioning; SCD = sickle cell disease; FSIQ = full scale IQ.

## Data Availability

No new data were created or analyzed in this study.
